# Neurological deterioration induced by sitting in patients after cervicothoracic posterior decompression with instrumented fusion surgery for ossification of the longitudinal ligament: two cases reports

**DOI:** 10.1186/s13104-015-1106-z

**Published:** 2015-04-09

**Authors:** Masao Koda, Chikato Mannoji, Taigo Inada, Koshiro Kamiya, Mitsutoshi Ota, Satoshi Maki, Kazuhisa Takahashi, Masashi Yamazaki, Masaaki Aramomi, Osamu Ikeda, Takeo Furuya

**Affiliations:** Departments of Orthopedic Surgery, Chiba University Graduate School of Medicine, 1-8-1, Inohana, Chuo-K, Chiba, 260-8670 Japan; Chiba Aoba Municipal Hospital, Chiba, Japan; University of Tsukuba, Tsukuba, Japan

**Keywords:** Ossification of the posterior longitudinal ligament, Posterior surgery, Instrumented fusion, Postoperative complication

## Abstract

**Background:**

We report on Japanese patients who showed neurological deterioration induced by sitting after cervicothoracic posterior decompression with instrumented fusion, but showed immediate neurological recovery after bed rest.

**Case Presentation:**

Patients showed incomplete paraparesis caused by the ossification of the posterior longitudinal ligament at uppermost thoracic spine. Cervicothoracic posterior decompression with instrumented fusion was performed. Postoperatively, the patients showed partial paraparesis when they were sitting. They showed rapid recovery from lower extremity paralysis upon lying down. After strict bed rest for one month, those patients showed no apparent development of paralysis during sitting.

**Conclusion:**

In patients with postoperative residual anterior spinal cord compression, micromotion might exacerbate neurological symptoms.

## Background

Ossification of the longitudinal ligament (OPLL) is one of anterior spinal cord compressive lesions at the uppermost thoracic spine, and is often treated using a posterior approach to decompression because of the anatomical complexity of the upper mediastinum [1]. OPLL patients showing local kyphosis often have a poor surgical outcome after posterior decompression surgery alone [2]. Concurrent instrumented posterior fusion is usually adopted as a stabilization procedure. The rationale for posterior decompression with instrumented fusion surgery (PDF) is to obtain neurological recovery by immediate stabilization of the spine, even if there is residual anterior compression following the procedure [3-6]. Here we report two cases that showed neurological deterioration induced by sitting after cervicothoracic PDF for OPLL, but showed immediate neurological recovery after bed rest.

## Case presentations

### Case 1

**Figure 1:**
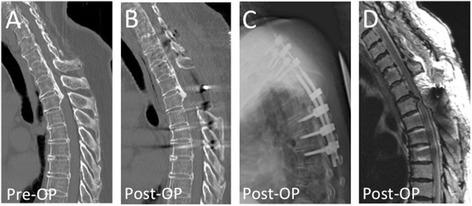
**Pre- and postoperative images of Case 1.** Pre-operative computed tomography image showed beak-shaped ossification of the posterior longitudinal ligament at T2/3 vertebral level **(A)**. Posterior decompression with instrumented fusion surgery at C7-T5 level was performed **(B-D)**. This patient showed partial paraparesis when she was sitting, but showed immediate neurological recovery after bed rest.

A 71-year-old Japanese woman complained of difficulty walking caused by OPLL at T2-3. C7–T5 PDF surgery was performed (Figure 1A, B, C). Three days after surgery, the patient showed partial paraparesis when she was sitting. Emergent CT and MRI showed no apparent abnormalities (Figure 1D). During those examinations, the patient gradually recovered from lower extremity paralysis. We ordered strict bed rest without sitting up for one month. Subsequently, the patient showed no apparent development of paralysis during sitting or using a wheelchair.

### Case 2

**Figure 2:**
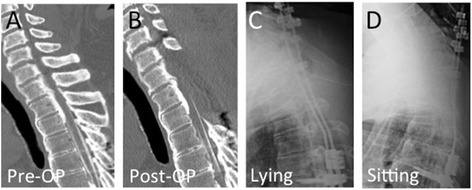
**Pre- and postoperative images of Case 2.** Pre-operative computed tomography image showed peak of ossification of the posterior longitudinal ligament at T1/2 vertebral level **(A)**. C3-T5 posterior decompression with instrumented fusion surgery was performed. The patient showed incomplete paralysis on sitting, but rapidly recovered after bed rest. An emergent computed tomographic-myelogram revealed no apparent abnormalities **(B)**. X-ray images obtained when the patient was lying **(C)** and sitting **(D)** showed no apparent motion between fused segments.

A 37-year-old Japanese man showed partial paraparesis because of OPLL at C3–T3 (worst at T1-2, Figure 2A). The patient underwent PDF surgery at C3–T5. The patient showed incomplete paralysis on sitting, but rapidly recovered after bed rest. An emergent CT-myelogram revealed no apparent abnormalities (Figure 2B). X-ray images obtained when the patient was sitting and lying showed no apparent motion between fused segments (C7-T4 angle was 4° in both lying and in sitting position, Figure 2C and D). We ordered strict bed rest for 3 weeks. Subsequently, the patient showed no apparent development of paralysis during sitting.

## Discussion

Points in common between the present patients were that there was an anterior spinal cord compressive lesion at the upper thoracic level, that there was residual anterior spinal cord compression after surgery and the cervical 3.5 mm diameter rods were used at the cervicothoracic region. Previous Japanese reports describing neurological deterioration induced by sitting after cervicothoracic posterior decompression with instrumented fusion for OPLL showed similarities with the present patients [7,8]. Both of the present cases showed rapid neurological deterioration with sitting, followed by rapid recovery on lying down. These lines of indirect evidence lead us to suggest that, even after PDF surgery, there is micromotion at the fused segments. In contrast, no mid- to lower-thoracic OPLL patients showed neurological deterioration at sitting after PDF surgery in our previous series [5,6].

The possible differences between the previous mid- to lower-thoracic OPLL series and the present cases are the difference in the region and the difference of rod diameter, tapered rod (4.5 mm thoracic and 3.5 cervical) for case 1 and 3.5 mm cervical rod for case 2. Upper thoracic spinal cord might be vulnerable for external force because of its specific blood supply [9]; therefore in patients with postoperative residual anterior spinal cord compression at upper thoracic region, the spinal cord might be more vulnerable for micromotion than in the patients of mid- to lower thoracic spinal level.

## Conclusion

In conclusion, we recommend surgeons be alert to the possibility of micromotion after cervicothoracic PDF surgery for OPLL. Thicker or more rigid rods for the PDF surgery may suppress this micromotion.

## Consent

Written informed consent was obtained from both of the patients for publication of this Case Report and any accompanying images. A copy of the written consent is available for review by the Editor-in-Chief of this journal.

## Abbreviations

OPLL: Ossification of the posterior longitudinal ligament
PDF: Ossification of the longitudinal ligament
CT: Computed tomography
MR: Magnetic resonance
